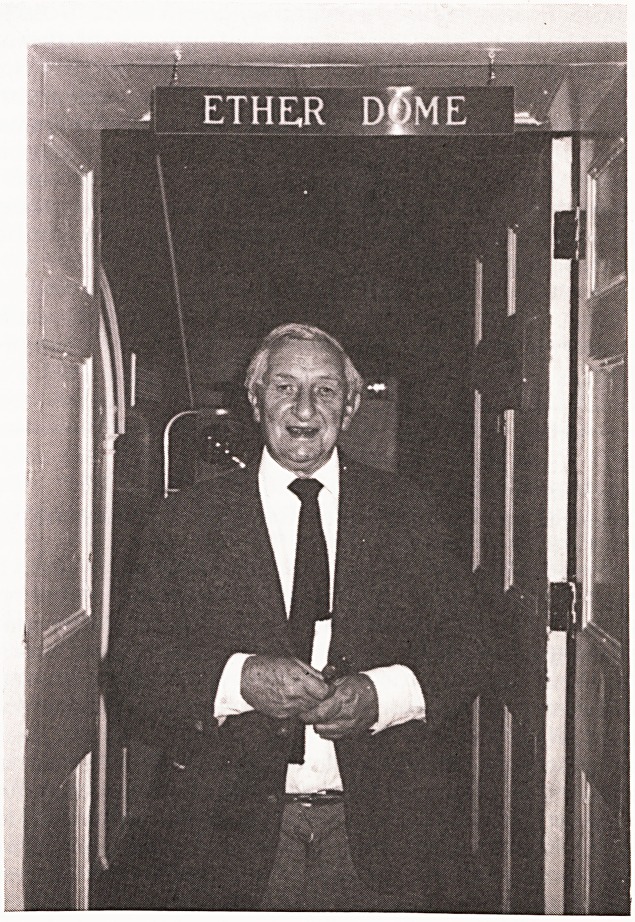# Dr Terence Steen

**Published:** 1987-05

**Authors:** 


### Obituary

### Terence Steen, MA, MB, BChir, FFARCS.

Dr Terence Ross Steen, formerly consultant anaesthetist at Southmead Hospital Bristol, died suddenly on March 20th, aged 68.

Dr Steen came from a strong medical family. His father, an Ulsterman who came to London to practice, his uncle, half brother, cousin and sister were all medical practitioners. He possessed a first rate intellect and a deep knowledge of English Literature, and with an unusually accurate and retentive memory could quote extensively. While at school at Clifton College, where he was a junior scholar, he won the English Essay Prize, an unusual if not unique achievement for anyone on the science side. He loved all sport and his personal qualities made him a natural leader, he became head of house, school prefect and captain of several teams.

From Clifton he went to Clare College, Hospital where he was President of the student's union. He qualified in 1943 with the M.B., B.Chir. Cambridge. He was appointed house physician and then house Surgeon at the London Hospital and in 1944 he joined the RAMC. His service included work as an anaesthetist to a Field Surgical Unit and General Hospitals in NW Europe. On returning to the UK he became Junior and then Senior Resident Anaesthetist at the London Hospital. In 1948 he passed the DA and moved to the West Dorset Hospital in Weymouth, combining anaesthetics with general practice. Here he joined his brother who was also a GP surgeon at the same hospital. In 1950 after two years at Weymouth he decided to return to full time hospital work and obtained an appointment as Anaesthetic Registrar at St George's Hospital, London. Later the same year he was appointed as senior registrar to the United Bristol Hospitals and in 1952 became Consultant Anaesthetist to Southmead Hospital Bristol. Here he remained until his retirement in 1980, except for a two year secondment to Mulago Hospital in Uganda from 1959 to 1961. He had the status of Lecturer in the Department of Surgery at Makerere College with the principle task of organising the anaesthetic services in Uganda and was one of two Anaesthetists (sometimes the only one) in the whole of that country. His most important duty was the training of "Medical Assistants" (superior male nurses) upon whom the anaesthetic service almost entirely depended. He remembered this period as one of the most useful in his life and was greatly saddened by the subsequent ruination of the country under Amin.

In 1967 Terence Steen was President of the Society of Anaesthetists of the South West Region. He was also Chairman of the Southmead Hospital Medical Staff Advisory Committee. He was a member of the Association of Anaesthetists and a Fellow of the Faculty of Anaesthetists of the RCS since 1954. He was also a member of the Medical Reading Society of which he was recently secretary and where his literary talents were much at home.

Terence was very highly regarded as an anaesthetist. Surgeons felt safe with his practical no-nonsense approach and patients remembered his reassuring kindness and total involvement. He understood the Art of Anaesthesia as well as being greatly knowledgable in the Science. Nevertheless he always preferred the simple and straightforward to the sophisticated techniques and was not one to press for new and expensive technology. For most of his life he preferred to ventilate a patient manually with a bag rather than use a mechanical ventilator. He felt it was safer and that he was more in touch with the patient, even though he forfeited the freedom to move around the theatre that the machine confers. He had an especial mastery in regional anaesthetic techniques which he used for poor risk patients and in the alleviation of postoperative pain. He was especially concerned with preventing 'awareness' in the paralysed patient. He bore a major responsibility for the anaesthetic service in the newly formed University Department of Obstetrics at Soutmead Hospital and pioneered the use of epidural anaesthesia in childbirth. He would spend hours helping his junior colleagues to prepare for their specialist exams. He took pains to teach them the use of open ether, an art which he believed should not be lost. He taught them they were'not just there to give an anaesthetic' but also the invaluable art of successfully running the list.

Terence had a great gift for writing humorous verse and could recall without effort most of the gems of Hilaire Belloc, Lewis Carroll and Edward Lear. He was a devotee of the great Dr Johnson who had a remark to fit every occasion; many was the time an apt quotation would be heard from the head of the operating table. Only recently he won first prize in a literary competition organised by the Medical Defence Union. It was required to construct a story linking '11 unlikely requests for advice drawn from the Nineteenth Century Annual Reports'. Not only did he succeed in producing a plausible story, but he did so in stanzas of limerick verse. The result was a brilliant and astonishing tour de force. (J. Med. Defence Union, 1987, Vol 3, 24.). Another example of his comic muse was the article 'Gardening, the great test of Health' (Bristol Med-Chi. J, 1986, 101, 66). The 'dangers of gardening' were a favourite subject for his humour; he proposed for gardeners a hymn with the refrain 'Protecting whereso'er they go, all those in hazard with the hoe'. He loved the broad humour of the old Music Hall and of the Cockney around the London Hospital. He was an expert in the Cockney rhyming slang and referred to the DA as the "Ta Ra Ra Boom". A joke was never far from his lips and yet no-one remembers him saying anything that was crude or unkind. He was a bibliophile with a vast collection of books and papers stored around the house on handsome shelves he had built himself.

Terence's family life was ideally happy. He met his wife Inge at the end of the War when he was attending an International Cultural Seminar in the Tirol to help restore harmony in a shattered Europe. Inge was also there as a student, they met and they married and the harmony has lasted a lifetime. Her father, a noted academic before the war was a known anti-Nazi and was imprisoned for three years in Dachau. He survived and on release was a national hero. He became Deputy Governor of the Tirol, a post he held until his death 30 years later. He gave Inge a beautiful house at Bach in the Tirol which has been their second home and where they have given generous hospitality to very many friends. Their three sons have each in their different ways been a source of happiness and pride.

M.G.W. and J.T.M.

## Figures and Tables

**Figure f1:**